# Free Treatment for Those Affected

**Published:** 1914-08-15

**Authors:** 


### LONDON PANEL COMMITTEE AND THE WAR.

### Free Treatment for those Affected.

A special meeting of the London Panel Committee was called to consider the following proposals: "That the Panel Committee is of opinion: (*a*) That the practitioners on the panel for the County of London should express their willingness to undertake free of charge the medical treatment of necessitous dependants of (i) reservists on their lists who have been called to the colours, and (ii) of insured persons on their lists who have become unemployed as a result of the war. (*b*) That in London steps should be taken to secure the medical treatment by practitioners on the panel of insured persons on the lists of those of their colleagues who are absent on military duty and who have been unable to make suitable arrangements; that the practitioners on the panel be so informed; and that the Secretary be instructed to take such steps as he may deem advisable in the matter."

The Chairman (Dr. H. J. Cardale), in moving the resolution, remarked that everyone could not fight under the colours, but this was a way in which those who remained at home could render service. He was sure every practitioner would be willing to do as the resolution proposed. Dr. J. A. Angus (vice-chairman) mentioned the question of drugs, and suggested that some means would have to be adopted to keep medicines prescribed under this resolution separate from those paid for by the Insurance Fund. Dr. Sasun thought practitioners should supply the drugs themselves and not trouble the chemists. An offer of this kind should be unconditional. Dr. Nundy agreed with Dr. Angus, and said he believed chemists would be willing to dispense at a discount of 33⅓ per cent.

Dr. H. H. Mills thought the doctors should undertake the cost of dispensing; the money could not come from the Insurance Fund, and doctors who did not dispense should make an arrangement with a chemist.

Dr. Angus said he was glad to see the feeling of the meeting and to withdraw his suggestion.

Dr. R. V. Donnellan hoped the Committee would not go too far in its patriotic desires. The resolution would press a great deal more severely upon practitioners in some districts than upon others. He wished the proposal could be extended so that they could work in conjunction with practitioners not on the panel, and a more equal distribution of the work be arrived at.

Dr. B. A. Richmond (Secretary to the Committee) said it was very desirable not to trespass on the capacity for loyalty of the non-panel section or interfere with their natural desire to render similar service. The Panel Committee could not speak for other than panel practitioners.

Dr. Claud Taylor hoped the profession would include persons who belonged to nations with whom we were at war. In consequence of the withdrawal of so many practitioners there would be wide gaps in the medical service in London, and he hoped doctors would not be encouraged to leave their work at home when there were probably sufficient men unattached to take up duties at the front. Dr. A. Reid agreed that practitioners should make no difference in respect of nationality in treating patients.

The resolution was approved, and it was decided to send a copy to all practitioners on the panel asking them to express their approval or otherwise.

The following resolution was also carried: "That this Committee appoint a sub-committee of six, with powers of co-option, to advise as to any arrangements suggested by the Government or any other body appointed to deal with the problem of sickness, unemployment, or destitution in the County of London which may result from the state of war."

There were appointed to serve on the sub-committee: Drs. Cardale, H. H. Mills, Lauriston Shaw, C. Taylor, R. V. Donnellan, and Ethel Bentham.

### The Shortage of Drugs.

The following letter was received from the Secretary of the Pharmaceutical Committee:

"In consequence of the present war a serious situation has arisen with regard to the supply of drugs in this country, and I am therefore directed to call your attention to the fact that many drugs, such as antipyrine, phenacetin, salicylates, etc., are made abroad under foreign patents, and others, such as bromides, are manufactured from raw materials which do not exist in this country. Therefore I may add that it has become necessary for wholesale houses to issue to chemists a notice to the effect that each order for drugs and chemicals will be modified, so that a fair distribution of the available stock is made amongst their customers. In order that all useful drugs may be available as long as possible for serious cases of illness and for casualties arising out of a state of war, it would be highly advantageous if the Panel Committee would exercise its influence jointly with the Pharmaceutical Committee for the purpose of husbanding the present supplies and resources, which are none too ample."

The Chairman mentioned that quinine was very short; so was sugar, and for that he suggested practitioners might use aqua chloroform. Potassium iodide was up; lint was 25 per cent. up. All those preparations should be used with the greatest economy consistent with efficiency. Dr. J. A. Angus moved: "That the practitioners on the panel be notified of the facts brought to the notice of the Panel Committee by the Pharmaceutical Committee, and be asked to regulate their prescribing as far as possible accordingly."

Dr. Lauriston E. Shaw remarked that at his hospital a list was being prepared for circulation amongst members of the staff of the drugs that had become extremely scarce. If practitioners put their minds to it they could save an enormous amount of money by resorting to less expensive substitutes. Liquid paraffin, which was an expensive luxury, had been prescribed in enormous quantities by everybody on the staff of Guy's, and now there had been substituted for it castor oil, which was quite as efficacious and not likely to be taken in such large quantities. (Laughter.)

Dr. R. V. Donnellan suggested that the Pharmacy Sub-Committee should issue a list of drugs of which there was a shortage, with the names of suitable equivalents, and the Chairman promised that this should be done. The resolution was then carried.

